# Association Between NOX2-Mediated Oxidative Stress, Low-Grade Endotoxemia, Hypoalbuminemia, and Clotting Activation in COVID-19

**DOI:** 10.3390/antiox13101260

**Published:** 2024-10-17

**Authors:** Roberto Carnevale, Cristina Nocella, Raffaella Marocco, Paola Zuccalà, Anna Carraro, Vittorio Picchio, Alessandra Oliva, Roberto Cangemi, Maria Claudia Miele, Massimiliano De Angelis, Francesca Cancelli, Giovanni Enrico Casciaro, Luca Cristiano, Pasquale Pignatelli, Giacomo Frati, Mario Venditti, Francesco Pugliese, Claudio Maria Mastroianni, Francesco Violi, Lorenzo Ridola, Cosmo Del Borgo, Silvia Palmerio, Emiliano Valenzi, Rita Carnevale, Domenico Alvaro, Miriam Lichtner, Vincenzo Cardinale

**Affiliations:** 1 Department of Medical-Surgical Sciences and Biotechnologies, Sapienza University of Rome, 04100 Latina, Italy; gianni.casciaro@gmail.com (G.E.C.); fraticello@inwind.it (G.F.); 2 IRCCS Neuromed, 86077 Pozzilli, Italy; vittorio.picchio@uniroma1.it; 3 Department of Clinical Internal, Anesthesiological and Cardiovascular Sciences, Sapienza University of Rome, 00161 Rome, Italy; cristina.nocella@uniroma1.it (C.N.); pasquale.pignatelli@uniroma1.it (P.P.); francesco.violi@uniroma1.it (F.V.); 4 Infectious Diseases Unit, Santa Maria (SM) Goretti Hospital, Sapienza University of Rome, 04100 Latina, Italy; raffaella.marrocco@uniroma1.it (R.M.); paola.zuccala@uniroma1.it (P.Z.); cosmo.delborgo@uniroma1.it (C.D.B.); 5 Department of Public Health and Infectious Diseases, Sapienza University of Rome, 00185 Rome, Italy; anna.carraro@uniroma1.it (A.C.); alessandra.oliva@uniroma1.it (A.O.); mariaclaudia.miele@uniroma1.it (M.C.M.); massimiliano.deangelis@uniroma1.it (M.D.A.); francesca.cancelli@uniroma1.it (F.C.); mario.venditti@uniroma1.it (M.V.); claudio.mastroianni@uniroma1.it (C.M.M.); miriam.lichtner@uniroma1.it (M.L.); 6 Department of Translational and Precision Medicine, Sapienza University of Rome, 00185 Rome, Italy; roberto.cangemi@uniroma1.it (R.C.); lorenzo.ridola@uniroma1.it (L.R.); domenico.alvaro@uniroma1.it (D.A.); vincenzo.cardinale@uniroma1.it (V.C.); 7 Helios Hospital West of Munich, 81241 Munich, Germany; dr.lucacristiano@gmail.com; 8 Department of General Surgery and Surgical Specialty, Sapienza University of Rome, 00161 Rome, Italy; f.pugliese@uniroma1.it; 9 Centro Ricerche Cliniche di Verona (CRC), 37134 Verona, Italy; palmerio.sil@gmail.com; 10 Department of Prevention, ASL Latina, 04011 Latina, Italy; emi.valenzi@gmail.com; 11 Corso di Laurea di I Livello in Infermieristica, Università Sapienza di Roma–Polo Pontino–Sede di Terracina, 04019 Terracina, Italy; rita.carnevale@uniroma1.it

**Keywords:** oxidative stress, NOX2 activation, albumin, D-dimer, gut permeability, low-grade endotoxemia, thrombosis

## Abstract

Low-grade endotoxemia by lipopolysaccharide (LPS) has been detected in COVID-19 and could favor thrombosis via eliciting a pro-inflammatory and pro-coagulant state. The aim of this study was to analyze the mechanism accounting for low-grade endotoxemia and its relationship with oxidative stress and clotting activation thrombosis in COVID-19. We measured serum levels of sNOX2-dp, zonulin, LPS, D-dimer, and albumin in 175 patients with COVID-19, classified as having or not acute respiratory distress syndrome (ARDS), and 50 healthy subjects. Baseline levels of sNOX2-dp, LPS, zonulin, D-dimer, albumin, and hs-CRP were significantly higher in COVID-19 compared to controls. In COVID-19 patients with ARDS, sNOX2-dp, LPS, zonulin, D-dimer, and hs-CRP were significantly higher compared to COVID-19 patients without ARDS. Conversely, concentration of albumin was lower in patients with ARDS compared with those without ARDS and inversely associated with LPS. In the COVID-19 cohort, the number of patients with ARDS progressively increased according to sNOX2-dp and LPS quartiles; a significant correlation between LPS and sNOX2-dp and LPS and D-dimer was detected in COVID-19. In a multivariable logistic regression model, LPS/albumin levels and D-dimer predicted thrombotic events. In COVID-19 patients, LPS is significantly associated with a hypercoagulation state and disease severity. In vitro, LPS can increase endothelial oxidative stress and coagulation biomarkers that were reduced by the treatment with albumin. In conclusion, impaired gut barrier permeability, increased NOX2 activation, and low serum albumin may account for low-grade endotoxemia and may be implicated in thrombotic events in COVID-19.

## 1. Introduction

Coronavirus disease 2019 (COVID-19) is a serious lung disease that may be complicated by severe acute respiratory syndrome (SARS-CoV-2) needing mechanical ventilation and intensive care unit treatment. Among the clinical features of COVID-19, thrombotic events in the venous and arterial are frequent complications that are associated with poor clinical outcomes [1,2,3,4,5,6]. Warning signs of thrombosis include essentially elevated markers of D-dimer, that is, expression of systemic fibrinolysis consequent to thrombin generation [7,8,9]; elevated markers of D-dimer have been consistently reported in COVID-19 patients and closely associated with thrombotic events and poor survival [10,11,12,13,14,15]. The mechanism accounting for hypercoagulation in COVID-19 has not been clarified but may include direct interaction of the virus or its components, such as spike protein, with cells devoted to activating coagulation systems such as endothelial cells, platelets, and leucocytes, or an abnormal response to virus injury with the production, for example, of cytokines [16] or oxidative stress that may in turn activate the clotting system. Patients with community-acquired pneumonia (CAP) display enhanced levels of lipopolysaccharides (LPS) coincidentally with an ongoing prothrombotic state, suggesting that low-grade endotoxemia may be implicated in the thrombotic complications occurring in CAP [17,18]. In addition, CAP patients also display enhanced levels of NOX2, one of the most important cellular producers of reactive oxidant species (ROS) [19]. Increasing evidence has demonstrated that dysregulation of ROS production can result in persistent inflammation and tissue damage, particularly in barrier sites such as the lung, reducing the body’s ability to clear viral infections [20]. Specifically, NOX2 can be activated during viral infections, resulting in the production of hydrogen peroxide that suppresses the antiviral response and favors viral replication [20,21].

Therefore, we speculated that NOX2-mediated oxidative stress and low-grade endotoxemia may be implicated also in COVID-19. Analysis of NOX2 and LPS in COVID-19 showed, in fact, that they are elevated compared to controls and associated with thrombosis, suggesting that LPS could promote a hypercoagulation state [22]. The biological plausibility of this hypothesis relies on an experimental model of thrombosis where low-grade endotoxemia enhanced thrombus growth via the LPS-specific receptor, i.e., Toll-like receptor 4 (TLR4) [23]. Previous studies [22,24,25] have linked higher intestinal translocation with severe cases of COVID-19, describing a higher abundance of bacterial components in the bloodstream compared to milder cases.

However, so far, the relationship between NOX2, LPS, and clotting activation has not been investigated in COVID-19, nor has it clarified the reason for low-grade endotoxemia.

Albumin is the most relevant protein in human blood, which possesses anti-inflammatory, antioxidant, and anticoagulant properties [26,27]. Also, albumin can neutralize LPS, preventing its toxic effects [28,29]. Previous studies showed that in COVID-19, serum albumin is seriously reduced during the acute phase of the disease and correlated with mortality and thrombotic events [30,31]. Therefore, the aim of this study was to explore the interplay between NOX2, LPS, and albumin and to assess if such interplay may be implicated in the hypercoagulation state of COVID-19. In addition, we would aim to verify if NOX2 and LPS could predict disease severity, especially ARDS.

## 2. Materials and Methods

### 2.1. Study Design and Population

This is an observational retrospective cohort multi-center study performed in Italian hospitals devoted to COVID-19 care. This study was performed in non-intensive care unit (ICU) medical wards. We enrolled consecutive patients, according to the inclusion/exclusion criteria from the cohort from Rome Hospitals and Latina Hospital.

In this study, we included adult (>18 years) patients with laboratory-confirmed COVID-19-related or SARS-CoV-2-related pneumonia, requiring or not mechanical ventilation, consecutively hospitalized from March 2020 to May 2020. COVID-19 was diagnosed based on the World Health Organization interim guidance. Patients were classified as having or not having acute respiratory distress syndrome (ARDS), which was defined as the acute onset of respiratory failure, bilateral infiltrates on chest radiograph, hypoxemia as defined by a PaO_2_/FiO_2_ ratio ≤ 200 mmHg, and no evidence of left atrial hypertension or a pulmonary capillary pressure < 18 mmHg (if measured) to rule out cardiogenic edema [32]. Healthy subjects matched for demographic characteristics and without acute infections were used as controls; they were recruited for routine screening for cardiovascular disease from the Division of I Clinica Medica, Atherothrombosis Center, Policlinico Umberto I, Rome.

The routine analysis included P/F ratio and hs-CRP executed within 48 h from the admission at the hospital. Ethical approval for this study was obtained from the Ethics Committee of Azienda Ospedaliera Universitaria Policlinico Umberto I (ID Prot. 6192) and was conducted in accordance with the Declaration of Helsinki.

### 2.2. sNOX2-dp Assay

sNOX2-dp concentration was measured with an ELISA method. Briefly, the assay is based on coating standards and serum samples into an ELISA 96-well plate overnight at 4 °C. After incubation, an anti-sNOX2dp–horseradish peroxidase (HRP) monoclonal antibody was added. The immobilized antibody–enzyme conjugates were quantified by adding tetramethylbenzidine (TMB) and a stop solution. The enzyme activity was measured spectrophotometrically at 450 nm; values were expressed as pg/mL. Intra-assay and inter-assay coefficients of variation were <10%.

### 2.3. Hydrogen Peroxide (H_2_O_2_) Production

Hydrogen peroxide (H_2_O_2_) concentrations were determined by a colorimetric assay according to the manufacturer’s instructions (Abcam, Cambridge, UK). Values were expressed as μM, and the intra- and inter-assay CV were both <10%.

### 2.4. LPS Assay

LPS levels were measured using a commercial ELISA kit (Cusabio, Wuhan, China) as previously described [23]. Values were expressed as picograms per milliliter; intra-assay and inter-assay coefficients of variation were <10%.

### 2.5. Zonulin Assay

Serum zonulin levels were measured using a commercial ELISA kit (Elabscience, Houston, TX, USA). The amount of zonulin was measured with a microplate autoreader at 450 nm. Values were expressed as nanograms per milliliter; both intra-assay and interassay coefficients of variation were <10%.

### 2.6. Plasma D-Dimer Assay

D-dimer levels in human plasma were measured using a commercial ELISA kit (Abcam, Cambridge, UK). Values were expressed as micrograms per milliliter; intra-assay and inter-assay coefficients of variation were <10%.

### 2.7. Albumin Assay

Albumin levels in human serum were measured using a commercial colorimetric assay kit (Abcam, Cambridge, UK). Values were expressed as grams per deciliter; intra-assay and inter-assay coefficients of variation were <10%.

### 2.8. Assessment of Intrahospital Ischemic and Embolic Events

The clinical course of the disease and its evolution were monitored during hospitalization. The appearance of new ischemic/embolic events was diagnosed as follows: (i) pulmonary thromboembolism by lung computed tomography scan [33]; (ii) myocardial infarction by electrocardiogram (ECG) changes associated with enhanced markers of cell necrosis [34]; (iii) acute brain ischemia by onset of new focal neurological signs and symptoms and confirmed, whenever possible, by nuclear magnetic resonance or computed tomography imaging [35]; and (iv) acute limb ischemia diagnosed according to American Heart Association guidelines [36].

### 2.9. In Vitro Study 

HUVEC (Human Umbilical Vein Endothelial Cells) were purchased from Lonza (Amboise, France) and cultured in EGM-2 complete medium. Sub-confluent cultures (2500/cm^2^) were expanded between passages 3–5. Cell morphology and growth were monitored by light microscopy and assessed by Trypan Blue. To stimulate the cultures, cells were starved overnight (endothelial basal medium (EBM), Lonza supplemented with 0.2% FBS). The following day, starvation media was withdrawn, and HUVEC were treated with or without LPS (160 pg/mL) in the presence or not of albumin (3–5 g/dL) [37]. As a negative control (NC), the same percentage of albumin–diluent (1% PBS) was used.

After stimulation, HUVEC-derived supernatants were stored at −80 °C until use. Each experiment was replicated five times. Supernatants were analyzed for sNOX2dp and H_2_O_2_ concentration as previously described. Factor VIII was also measured by ELISA used according to the manufacturer’s guidelines (Lifespan Bioscience, Seattle, WA, USA). The values of the release of factor VIII were expressed as U/dL. Inter- and intra-assay coefficients of variations were <10%.

### 2.10. Sample Size Calculation

We calculated the minimum sample size with respect to a two-tailed one-sample Student’s t-test, considering (i) a relevant difference in LPS of 20 pg/mL; (ii) a standard deviation of paired differences of 30 pg/mL; (iii) a type I error rate (α) of 0.05; and (iv) a power (1 − β) of 0.90. This yielded a required sample size of n = 49 per group. The expected LPS serum levels were conservatively estimated based on previously published data [22].

### 2.11. Statistical Analysis

All continuous variables were tested for normality with the Shapiro–Wilk test. Continuous variables with normal distribution were reported as mean ± standard deviation (SD), non-parametric variables as median and interquartile range (IQR). Between-groups comparisons were performed using an unpaired *T* test for normally distributed variables and using an appropriate non-parametric test for non-normally distributed variables (Mann–Whitney *U* test). Correlations were performed by Spearman’s rank correlation coefficient and described as Rs.

The prediction roles of serum LPS, zonulin, hs-CRP, and D-dimer were evaluated by means of area under the curve (AUC) for the receiver operating characteristic curve for predicting incident ARDS.

The bivariate and multivariate effects of prognostic factors on ARDS occurrence in COVID-19 patients were also assessed by means of logistic regression models. Wald confidence intervals and tests for odds ratios and adjusted odds ratios were computed based on the estimated standard errors. The stochastic level of entry into the multivariable model was set at 0.10.

Only *p* values < 0.05 were considered statistically significant. All tests were 2-tailed, and analyses were performed using computer software packages (IBM SPSS Statistics, ver. 27).

## 3. Results

Clinical characteristics of patients with COVID-19 and controls are reported in Table 1.

No significant differences were present in age, sex, body mass index, or smoking habit between patients and controls. Conversely, significant differences were present for prevalence of arterial hypertension, diabetes, atrial fibrillation, and chronic obstructive pulmonary disease (COPD) between patients and controls. As expected, COVID-19 patients with ARDS showed an increased prevalence of COPD, atrial fibrillation, and routine markers of inflammation and hypercoagulation.

**Figure 1:**
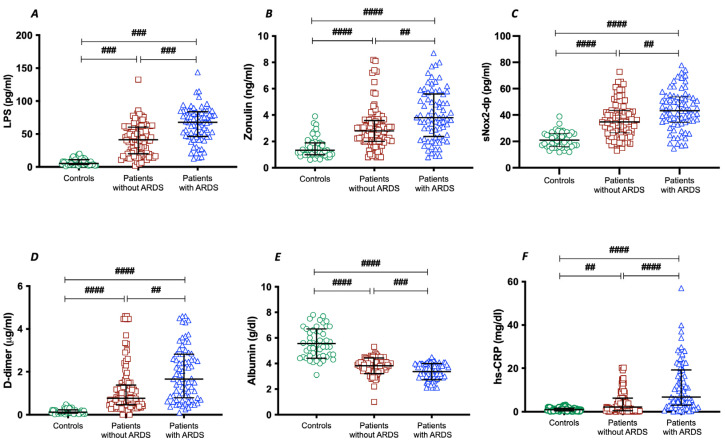
LPS (**A**), zonulin (**B**), sNOX2-dp (**C**), D-dimer (**D**), albumin (**E**), and hs-CRP (**F**) concentrations in COVID-19 patients without ARDS (n = 100), with ARDS (n = 75), and controls (n = 50). Data are expressed as median and interquartile range #### < 0.0001; ### < 0.001; ## < 0.01 non-parametric test (Kruskal–Wallis one-way ANOVA).

To assess the importance of gut permeability in the development of COVID-19 severity, we analyzed two markers of intestinal barrier dysfunction, such as LPS and zonulin. Serum LPS and zonulin were higher in patients with COVID-19 than in control subjects (Table 1); in COVID-19 patients with ARDS, LPS and zonulin were significantly higher compared to COVID-19 patients without ARDS (Table 1 and Figure 1A,B).

To analyze the interplay between oxidative stress and COVID-19 severity, we analyzed NOX2 levels in patients with and without ARDS. Significant differences were found in baseline levels of sNOX2-dp between controls and COVID-19 patients (Table 1 and Figure 1C). The levels of sNOX2-dp were higher in patients with ARDS compared with those without ARDS (Table 1 and Figure 1C). 

In addition, to analyze the interplay between coagulation and inflammation and COVID-19 severity, we analyzed levels of D-dimer, albumin, and hs-CRP in patients with and without ARDS. Significant differences were found in baseline levels of D-dimer, albumin, and hs-CRP between controls and COVID-19 patients (Table 1 and Figure 1D–F). Among COVID-19, serum concentrations of D-dimer and hs-CRP were higher in patients with ARDS (Table 1 and Figure 1D,F). Conversely, the serum concentration of albumin was lower in patients with ARDS compared with those without ARDS (Table 1 and Figure 1E).

**Figure 2:**
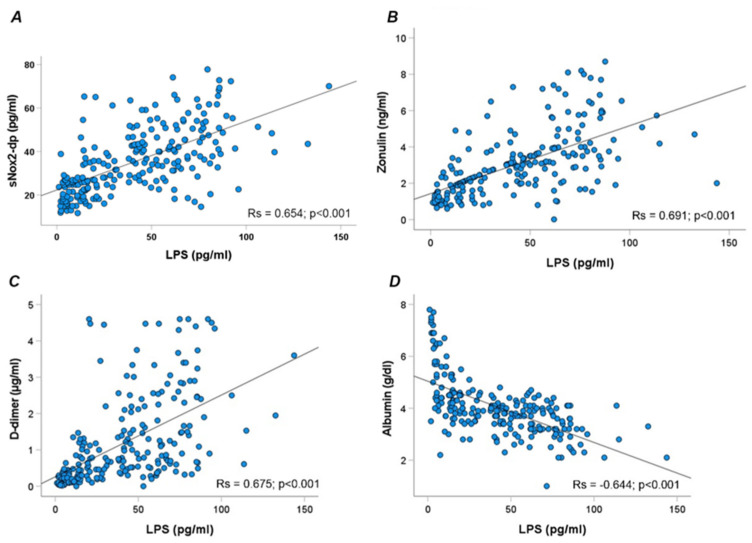
Scatter plots showing significant (two-tailed) spearman positive correlation of sNOX2-dp (**A**), zonulin (**B**), D-dimer (**C**), and albumin (**D**) in horizontal vs. vertical directions of LPS concentration.

Overall, LPS correlated directly with sNOX2dp (Rs = 0.654; *p* < 0.001), zonulin (Rs = 0.691; *p* < 0.001), D-dimer (Rs = 0.675; *p* < 0.001), and inversely with albumin (Rs = −0.644; *p* < 0.001) (Figure 2A–D).

Moreover, NOX2 correlated directly with D-dimer (Rs = 0.525; *p* < 0.001) and, inversely, with albumin (Rs = −0.443; *p* < 0.001).

**Figure 3:**
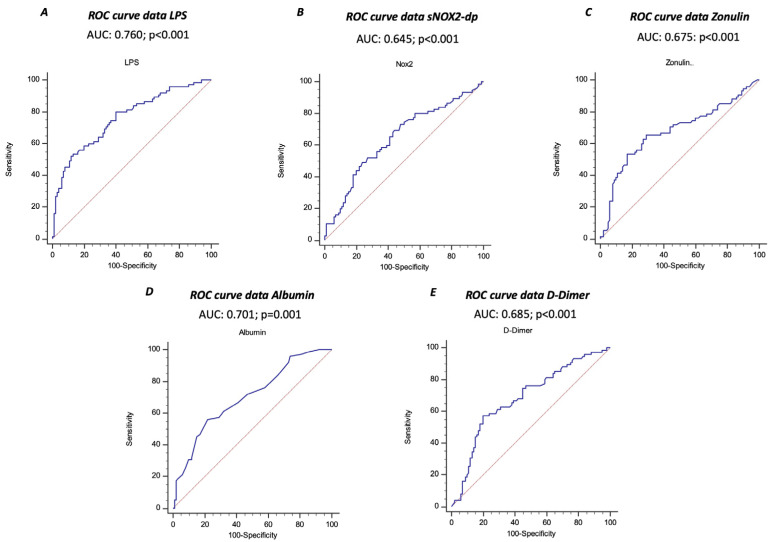
Receiver operating characteristic (ROC) curves (blu lines) of LPS (**A**), sNOX2-dp (**B**), zonulin (**C**), albumin (**D**), and D-dimer (**E**) against prediction of ARDS. Dotted red line represents AUC of 0.5.

The ROC curve analyses showed that sNOX2-dp, LPS, zonulin, albumin, and D-dimer predicted ARDS, with LPS showing the highest AUC (AUC for LPS: 0.760; 95% CI: 0.690–0.821; AUC for NOX2: 0.645; 95% CI: 0.562–0.728; AUC for zonulin: 0.675; 95% CI: 0.600–0.744; AUC for albumin 0.701; 95% CI: 0.627–0.767; AUC for D-dimer 0.685; 95% CI: 0.610–0.753) (Figure 3A–E).

**Figure 4:**
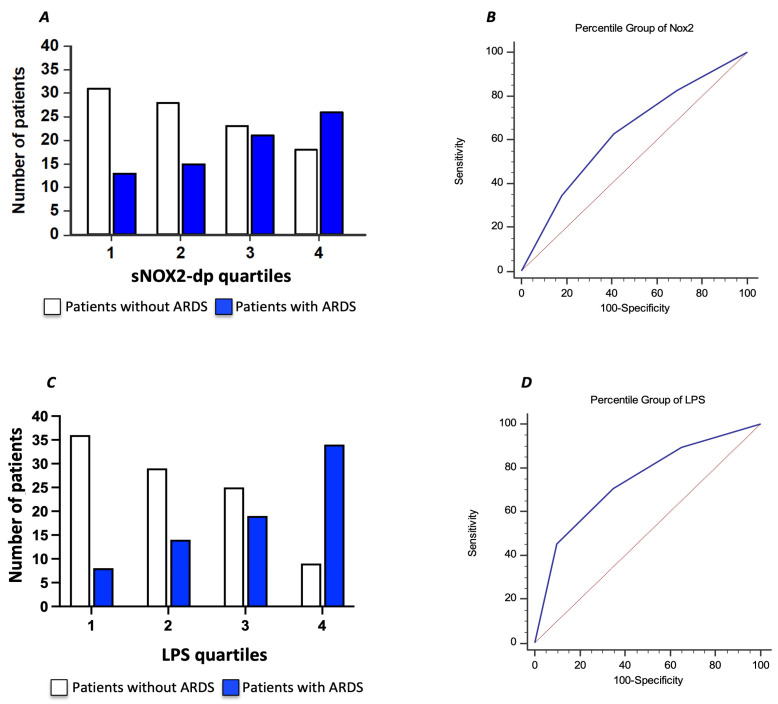
Relationship between serum sNOX2-dp and ARDS in COVID-19 patients, dividing the COVID-19 cohort according to sNOX2-dp quartiles (**A**). Area under the curve (AUC) of sNOX2-dp quartiles (Blu line) (**B**). Relationship between serum LPS and ARDS in COVID-19 patients, dividing the COVID-19 cohort according to LPS quartiles (**C**). Area under the curve (AUC) of LPS quartiles (Blu line) (**D**). Dotted red lines in B and D represent AUC of 0.5.

To further characterize the relationship between serum sNOX2-dp and ARDS in COVID-19 patients, we divided the COVID-19 cohort according to sNOX2-dp quartiles (I quartile, n = 44; sNOX2-dp ≤ 28 pg/mL; II quartile, n = 43; sNOX2-dp > 28 and ≤38.9 pg/mL; III quartile, n = 44; sNOX2-dp > 38.9 and ≤50.2 pg/mL; IV quartile, n = 44; sNOX2-dp > 50.2 pg/mL). The number of patients with ARDS progressively increased between the first and fourth quartiles (29%, 35%, 48%, and 59%, respectively; *p* = 0.024) (Figure 4A). The AUC of NOX2 quartiles was 0.630 (95% CI: 0.554–0.701), with values ≥ III quartiles predicting ARDS with a sensitivity of 63% and a specificity of 59% (Figure 4B).

Moreover, we characterized the relationship between serum LPS and ARDS in COVID-19 patients by dividing the COVID-19 cohort according to LPS quartiles (I quartile, n = 43; LPS ≤ 29.4 pg/mL; II quartile, n = 44; LPS > 29.4 and ≤50.3 pg/mL; III quartile, n = 44; LPS > 50.3 and ≤71.7 pg/mL; IV quartile, n = 44; LPS > 71.7 pg/mL). The number of patients with ARDS progressively increased between the first and fourth quartiles (19%, 32%, 43%, and 77%, respectively; *p* < 0.001) (Figure 4C). The AUC of LPS quartiles was 0.739 (95% CI: 0.667–0.802), with values ≥ III quartiles predicting ARDS with a sensitivity of 70.7% and a specificity of 65% (Figure 4D).

Finally, a multivariable logistic regression analysis showed that the IV quartile of LPS was independently associated with an increased risk of ARDS, together with low albumin serum levels and an increased hs-CRP, after adjusting for NOX2, D-dimer, age, sex, and comorbidities (Table 2).

During a median follow-up of 18 days, 21 patients experienced thrombotic events, 15 with ARDS and 6 without ARDS. Among the 75 patients with ARDS, 15 patients (20%) experienced thrombotic events in the arterial (n = 7) and venous circulation (n = 8) (interquartile range 11–27 days). Patients without ARDS (n = 100) had six (6%) (n = 6) thrombotic events (in the arterial n = 2 and venous circulation n = 4).

Of note, patients who experienced a thrombotic event showed higher levels of LPS than patients without thrombotic events (75.5 [52.2–85.1] vs. 46.4 [26.3–67.6] pg/mL; *p* < 0.001). In particular, the number of thrombotic events progressively increased from the I to the IV quartile of serum LPS (2% in the I, 9% in the II, 9% in the III, and 27% in the IV quartile, *p* < 0.001). Univariate logistic regression analyses showed that increased levels of LPS (OR: 1.037; 95% CI: 1.017–1.057; *p* < 0.001) and D-dimer (OR: 2.239; 95% CI: 1.1569–3.194; *p* < 0.001) and decreased levels of albumin (OR: 0.350; 95% CI: 0.178–0.688; *p* = 0.002) were associated with increased risk of thrombotic events. 

**Figure 5:**
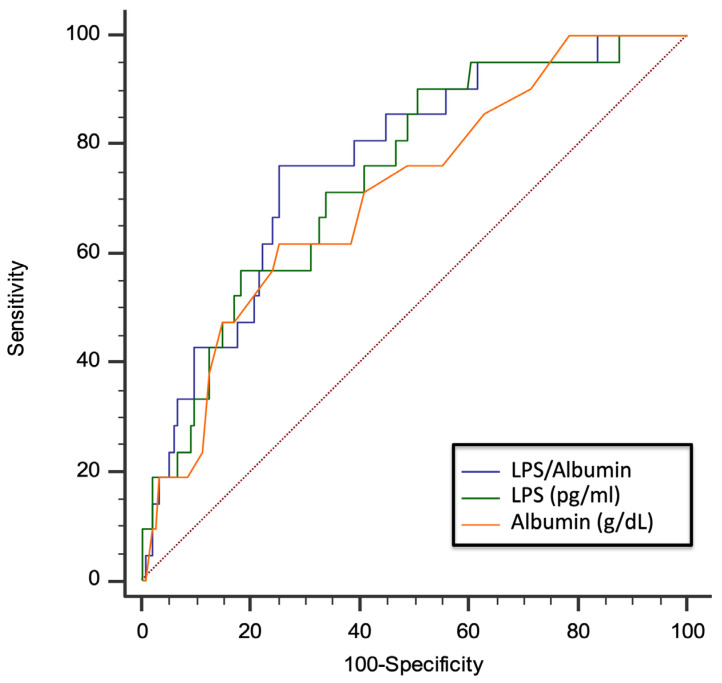
AUC of LPS/albumin ratio, LPS, and albumin for prediction of thrombotic events in COVID-19 patients. Dotted red line represents AUC of 0.5.

Due to the functional interplay between LPS and albumin, we evaluated the role of the LPS/albumin ratio as a predictor of thrombotic events. In a multivariable logistic regression model, both LPS/albumin levels (OR: 1.045; 95% CI: 1.005–1.087; *p* = 0.029) and D-dimer (OR: 2.002; 95% CI: 1.369–2.926; *p* < 0.001) predicted thrombotic events. The ROC curve analyses showed that the LPS/albumin ratio showed a higher AUC than LPS albumin alone (LPS/albumin AUC: 0.768; 95% CI: 0.699 to 0.829; *p* < 0.0001; LPS AUC: 0.745; 95% CI: 0. 0.673 to 0.808; *p* < 0.0001; albumin AUC: 0.712; 95% CI: 0. 0.638 to 0.778; *p* = 0.0003) (Figure 5).

In particular, the LPS/albumin ratio > 18.5 showed a sensitivity of 76.2% and a specificity of 74.7% to detect a thrombotic event. Interestingly, overall, the correlation between the LPS/albumin ratio and D-dimer was stronger (Rs = 0.687; *p* < 0.001) than the correlation between the correlation between LPS and D-dimer (Rs = 0.675; *p* < 0.001) and albumin and D-dimer (−0.596; *p* < 0.001). Dotted red line represents AUC of 0.5.

### In Vitro Study

**Figure 6:**
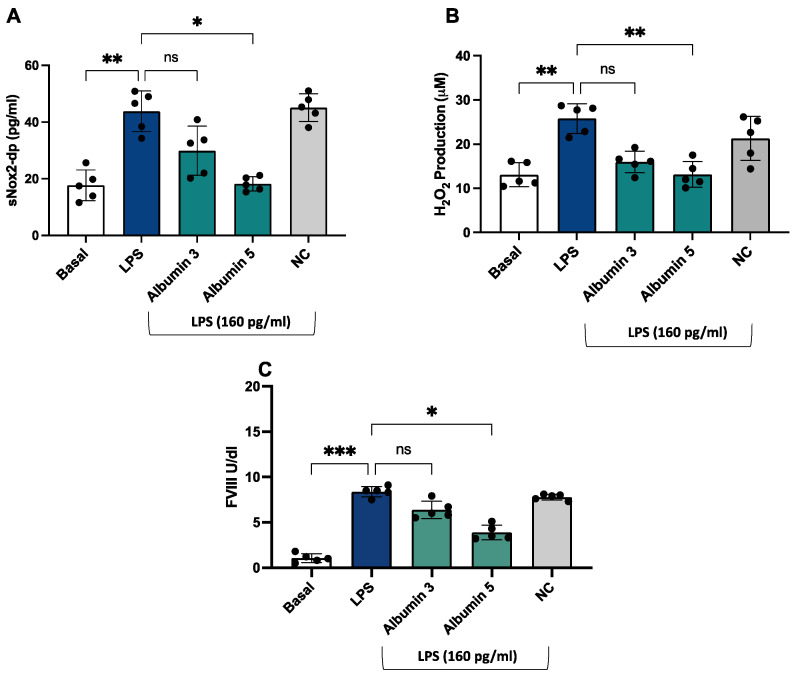
Albumin reduced LPS-mediated sNOX2-dp release, H_2_O_2_ production, and FVIII release in HUVEC. sNOX2-dp (**A**) and H_2_O_2_ (**B**) and FVIII (**C**) in HUVEC incubated with or without LPS (160 pg/mL) in the presence or not of albumin 3 and 5 g/dL or negative control (NC). Experiments were performed in five separate sets of HUVEC. Data are expressed as mean ± SD; *** *p* < 0.001; ** *p* < 0.01; * *p* < 0.05 values were calculated using an ANOVA non-parametric test.

After stimulation with LPS (160 pg/mL), we observed an increase in sNOX2dp, H_2_O_2_ production, and FVIII (Figure 6A–C) compared to unstimulated cells.

Albumin-treated endothelial cells before the stimulation with LPS showed a significant decrease in NOX2 activation (Figure 6A), H_2_O_2_ production (Figure 6B), and FVIII (Figure 6C) compared to LPS-stimulated cells; this effect was only evident at concentrations of 5 g/dL.

## 4. Discussion

The main findings of the present study show that (1) LPS and oxidative stress are significantly associated with a hypercoagulation state; (2) LPS and NOX2 are inversely associated with serum albumin; (3) LPS and NOX2 are significantly associated with disease severity; and (4) LPS/albumin ration significantly predicts thrombotic events.

LPS is a glycolipid component of the outer membrane of Gram-negative gut bacteria and is composed of carbohydrates and a portion of lipid A [38]. LPS may increase into systemic circulation depending upon the diet typology; after a high-fat-rich diet, its elevation is associated with enhanced intestinal biosynthesis of apolipoprotein B48, which serves to transport chylomicrons in the peripheral circulation [39]. Gut dysbiosis and/or impaired gut barrier dysfunction are key factors in determining LPS translocation into systemic circulation with ensuing low-grade endotoxemia and systemic inflammation [39]. In addition to metabolic diseases, systemic infections may contribute to gut dysbiosis and low-grade endotoxemia, as shown in patients with community-acquired pneumonia and more recently by COVID-19 [39]. Previous studies suggested that virus binding and entry into human cells occurs through the angiotensin-converting enzyme 2 (ACE2) receptor, which also localizes in the gastrointestinal tube [40]. Therefore, SARS-CoV-2 can invade and propagate in intestinal epithelial cells, weakening the mechanical barrier [41]. Moreover, the interaction between SARS-CoV-2 and the ACE2 receptor induces deep alterations to the intestinal microflora at phylogenetic and metabolomic levels [41].

Thus, the present study supports and extends a previous study from our group showing that endotoxemia is detectable in the early phase of COVID-19 disease with a significant association with D-dimer, suggesting that LPS contributes to the hypercoagulation state of COVID-19 patients. As above outlined, the increase in LPS in patients with pneumonia may depend on several mechanisms, including gut dysbiosis and/or impaired gut barrier dysfunction secondary to systemic infection or inflammation. Our previous data, which are confirmed by the present study, suggest a role for gut barrier dysfunction as a mechanism accounting for LPS translocation into systemic circulation, as indicated by the significant increase in zonulin, an indirect marker of gut barrier dysfunction [39], and its correlation with LPS. However, impaired gut permeability is not the only mechanism accounting for endotoxemia. Thus, the reduction in albumin may also play a role, as albumin serves to blunt LPS, hindering its toxic effects. Among the functions of albumin, its activity as an acute reactant protein largely explains its serum reduction during the acute phase of the disease [42]. This change may have deleterious clinical effects in several ways, “in primis”, by favoring LPS toxicity contributing to the systemic inflammation via LPS-induced overproduction of cytokines such as interleukin 6 or TNF-alpha [43].

Previous reports showed that LPS may elicit a hypercoagulation state acting at the level of several cell lines, such as platelets, leucocytes, and endothelial cells, that upon activation are involved in thrombin generation via NOX2-related platelet activation, macrophage Tissue Factor overexpression and factor VIII, and von Willebrand secretion by endothelial cells [39,44]. Therefore, oxidative stress leads to the dysregulation of coagulation and fibrinolysis processes, increasing the risk of thrombus formation [45]. According to our previous studies, we show significant differences between controls and COVID-19 patients in NOX2 levels that were higher according to COVID-19 severity. Moreover, we found a significant association between sNOX2-dp and D-dimer, suggesting a role of NOX2 in eliciting the hypercoagulation state in these patients.

The clinical consequence of this phenomenon is the worsening of systemic inflammation with a negative impact on infection progression; in accordance with this hypothesis is the significant association between LPS and COVID-19 severity, i.e., higher was LPS and more frequent was the occurrence of ARDS. The reduction in serum albumin is also important in the context of hypercoagulation of COVID-19. Thus, albumin possesses anticoagulant and antiplatelet effects by inhibiting the liver biosynthesis of coagulation factors or reducing the platelet biosynthesis of eicosanoids [46,47]; experimental studies in humans demonstrated that albumin supplementation exerts antiplatelet and anticoagulant effects [27,48,49]. Thus, the concomitant reduction in albumin along with the increase in LPS is a negative combination of factors that strongly contribute to enhanced thrombin generation and hypercoagulation and eventually higher thrombotic risk. To investigate this issue, we analyzed the impact of both variables on the thrombotic risk of our patients and found that the LPS/albumin ratio was more predictive of thrombotic risk than the single variable. The combined changes of LPS and albumin could seriously influence thrombotic outcomes in COVID-19.

Finally, we conducted an in vitro study to confirm the role of the LPS/NOX2 axis in hypercoagulation and the effect of albumin. LPS increases endothelial oxidative stress and coagulation biomarkers that were reduced in the presence of albumin (5 g/dL).

This study has implications. Reduction in LPS or Reduction in LPS could contribute to lower systemic inflammation and hypercoagulation state; thereby, further study should be addressed to modulate these two variables in COVID-19. A preliminary study with albumin supplementation showed a significant reduction in D-dimer, but the sample size as well as the study methodology did not allow a definite conclusion [49].

Our results could also be applied to several clinical conditions where endotoxemia and oxidative stress have been recognized as risk factors. Indeed, low-grade endotoxemia and enhanced oxidative stress have been the most widely investigated as they are associated with many inflammation-driven conditions, including atherosclerotic cardiovascular diseases, obesity, liver diseases, and diabetes [50,51].

Finally, in this context, measuring oxidative stress levels by NOX2 could help monitor and moderate the impact of LPS-related damage.

## 5. Limitations

This study has some limitations that warrant acknowledgment. Firstly, the sample size was relatively small, considering the numerous interactions observed among the different biomarkers analyzed. A larger study is warranted to further explore the relationships between LPS, NOX2, zonulin, and ARDS in this context. Some AUCs, particularly that of sNOX2dp in predicting ARDS, were not notably high compared to those for LPS levels. The reasons for these differing associations were not thoroughly investigated. It is likely that endotoxemia increases the risk of ARDS through multiple mechanisms, only partially involving enhanced NOX2-mediated oxidative stress.

We found an increase in circulating levels of zonulin, suggesting dysfunctionality of the gut barrier. However, further studies are necessary to investigate if factors intrinsic and/or extrinsic to gut microbiota are involved in low-grade endotoxemia in COVID-19.

Finally, we did not provide experimental evidence that the combination of high LPS and low albumin enhances the activation of intrinsic and/or extrinsic coagulation pathways more than a single variable; thereby, further study is necessary to explore this issue.

## 6. Conclusions

Patients with COVID-19 show a simultaneous increase in LPS and NOX2 activity associated with a reduction in albumin that may contribute to a hypercoagulation state and eventually increase the thrombotic risk. Interventional studies to lower LPS, NOX2 activity, or increase albumin are warranted to assess if this approach may reduce the thrombotic risk.

## Figures and Tables

**Table 1:** Characteristics of control subjects and COVID-19 patients.

Variable	Controls	COVID-19 Patients	*p*	Patients Without ARDS	Patients with ARDS	*p*
N	50	175		100	75	
Age (years) ^a^	65.7 ± 12.7	64.9 ± 16.0	0.720	64.6 ± 15.9	65.2 ± 16.3	0.804
Female sex (%)	34	34	0.970	40	27	0.066
BMI ^a^	26.6 ± 3.7	26.9 ± 3.6	0.701	26.0 ± 3.5	27.8 ± 3.5	0.087
Smokers	4	5	0.647	9	5	0.360
Hypertension (%)	0	27	<0.001	24	32	0.240
COPD (%)	0	10	0.022	15	4	0.006
Atrial fibrillation (%)	0	5	0.124	7	0.75	0.076
Diabetes (%)	0	18	0.001	19	16	0.607
ACE-inhibitors (%)	0	25	<0.001	20	31	0.105
Thrombotic events	0	21	<0.001	6	15	0.004
HS-CRP (mg/dL) ^b^	1 [0.3–1.5]	3.8 [1.1–10.8]	<0.001	2.1 [0.5–6.2]	6.7 [3.0–19.2]	<0.001
D-dimer (µg/mL) ^b^	0.12 [0.07–0.23]	1.09 [0.54–2.25]	<0.001	0.77 [0.46–1.38]	1.66 [0.79–2.81]	<0.001
Albumin (g/dL) ^a^	5.56 ± 1.14	3.62 ± 0.66	<0.001	3.82 ± 0.61	3.36 ± 0.63	<0.001
Zonulin (ng/mL) ^b^	1.33 [0.98–1.88]	3.10 [2.10–4.22]	<0.001	2.79 [2.0–3.54]	3.8 [2.37–5.60]	<0.001
LPS (pg/mL) ^b^	6.0 [3.7–11.7]	50.3 [29.4–71.7]	<0.001	41.5 [20.0–60.5]	67.7 [46.3–83.9]	<0.001
sNOX2-dp (pg/mL) ^b^	21.9 [16.0–26.1]	38.9 [28.0–50.2]	<0.001	34.8 [26.5–43.5]	43.2 [34.5–53.8]	0.001

ARDS, acute distress respiratory syndrome; BMI, body mass index; COPD, chronic obstructive pulmonary disease. ^a^ Data are expressed as mean values ± standard deviation (SD). ^b^ Data are expressed as median and interquartile range.

**Table 2:** Logistic regression analysis: predictors of ARDS in COVID-19 patients.

Variables	OR	95% CI		*p*
II quartile LPS vs. I	1.852	0.660	5.202	0.242
III quartile LPS vs. I	2.158	0.772	6.035	0.143
VI quartile LPS vs. I	6.819	2.231	20.843	0.001
Albumin	0.520	0.280	0.966	0.039
hs-CRP	1.079	1.023	1.138	0.005

## Data Availability

The data analyzed in this article will be shared upon reasonable request from the corresponding author.
